# Successful resolution of stromal keratitis and uveitis using canakinumab in a patient with chronic infantile neurologic, cutaneous, and articular syndrome: a case study

**DOI:** 10.1186/s12348-015-0065-9

**Published:** 2015-11-21

**Authors:** Masayuki Hirano, Jiro Seguchi, Masahiro Yamamura, Akiko Narita, Hirotaka Okanobu, Ryuta Nishikomori, Toshio Heike, Mio Hosokawa, Yuki Morizane, Fumio Shiraga

**Affiliations:** Department of Ophthalmology, Okayama University Graduate School of Medicine, Dentistry and Pharmaceutical Sciences, Okayama, Japan; Department of Ophthalmology, Okayama Saiseikai General Hospital, Okayama, Japan; Center for Rheumatology, Okayama Saiseikai General Hospital, Okayama, Japan; Department of Ophthalmology, Kurashiki Medical Center, Kurashiki, Japan; Department of Pediatrics, Kyoto University Graduate School of Medicine, Kyoto, Japan

**Keywords:** Cryopyrin-associated periodic syndrome, Chronic infantile neurologic, Cutaneous and articular/neonatal-onset multisystem inflammatory disease syndrome, Canakinumab, Stromal keratitis, Uveitis

## Abstract

**Background:**

Cryopyrin-associated periodic syndrome (CAPS) is a group of rare autoinflammatory diseases, and of these, chronic infantile neurologic, cutaneous, and articular/neonatal-onset multisystem inflammatory disease (CINCA/NOMID) syndrome has the most severe phenotype. Canakinumab, a monoclonal antibody that targets interleukin-1β, has been shown to be an effective treatment for resolving systemic inflammation. However, its efficacy for treating ophthalmic symptoms of this disorder remains unclear.

**Findings:**

A 64-year-old female reported episodes of nonpruritic urticaria, fever, aseptic meningitis, and bilateral sensorineural deafness. Her son had experienced similar symptoms. She was initially referred for ophthalmologic treatment for an infectious corneal ulcer. Examination of her right eye by slit lamp biomicroscopy showed diffuse conjunctival injection, corneal infiltrates, a corneal ulcer, and hypopyon. She was therefore treated aggressively with topical and systemic antibiotics in addition to antifungal medications. However, this was ineffective. Genetic analysis detected the heterozygous germline p.Asp303Asn mutation in the *NLRP3* gene in both our patient and her son. She was therefore diagnosed with CINCA/NOMID syndrome based on her clinical manifestations. All of the patient’s physical and ophthalmic symptoms were resolved within a few days after the initiation of canakinumab treatment. During an 18-month follow-up period, no adverse events or severe infections were observed.

**Conclusions:**

Our case report indicates that canakinumab is effective not only for the treatment of systemic inflammation but also for treating ophthalmic involvement, such as recurrent stromal keratitis and anterior uveitis.

## Findings

### Background

Cryopyrin-associated periodic syndrome (CAPS) is an autoinflammatory disorder caused by heterozygous mutations in the *NLRP3* gene and include three distinct conditions, namely familial cold autoinflammatory syndrome, Muckle-Wells syndrome, and chronic infantile neurologic, cutaneous, and articular (also known as neonatal-onset multisystem inflammatory disease) (chronic infantile neurologic, cutaneous, and articular/neonatal-onset multisystem inflammatory disease (CINCA/NOMID)) syndrome [1–5]. Gain-of-function mutations in *NLRP3* result in the excessive production of the potent proinflammatory cytokine interleukin-1β (IL-1β), thereby evoking the autoinflammatory manifestations of CAPS [6–8]. CINCA/NOMID syndrome is the most severe CAPS phenotype [9, 10]. The eye manifestations are pleiotropic in patients with CINCA/NOMID syndrome, with inflammation involving the cornea, sclera, anterior chamber, vitreous, retina, and optic disc [11]. Canakinumab, a fully humanized monoclonal antibody that selectively blocks IL-1β, has been shown to be an effective treatment for CINCA/NOMID syndrome [12–14]. However, the efficacy of canakinumab in treating its ophthalmic manifestations remains unclear.

Here, we describe a case of an adult female with CINCA/NOMID syndrome-related stromal keratitis and uveitis, which was successfully treated with canakinumab.

### Case presentation

A 64-year-old female was referred to our department for the treatment of an infectious corneal ulcer. She reported experiencing daily episodes of urticaria-like rashes, fevers, and arthralgia since birth. She also had a history of meningitis, which showed no evidence of infection and did not respond to antibiotic treatment, and bilateral sensorineural deafness since high school. The patient had been evaluated for collagen diseases many times at other hospitals, but no disease-specific autoantibodies had been detected. Her detailed ophthalmic history was unclear. Her family history was significant in that her son had displayed similar symptoms from birth that had been treated with corticosteroids and immunosuppressants.

**Fig. 1:**
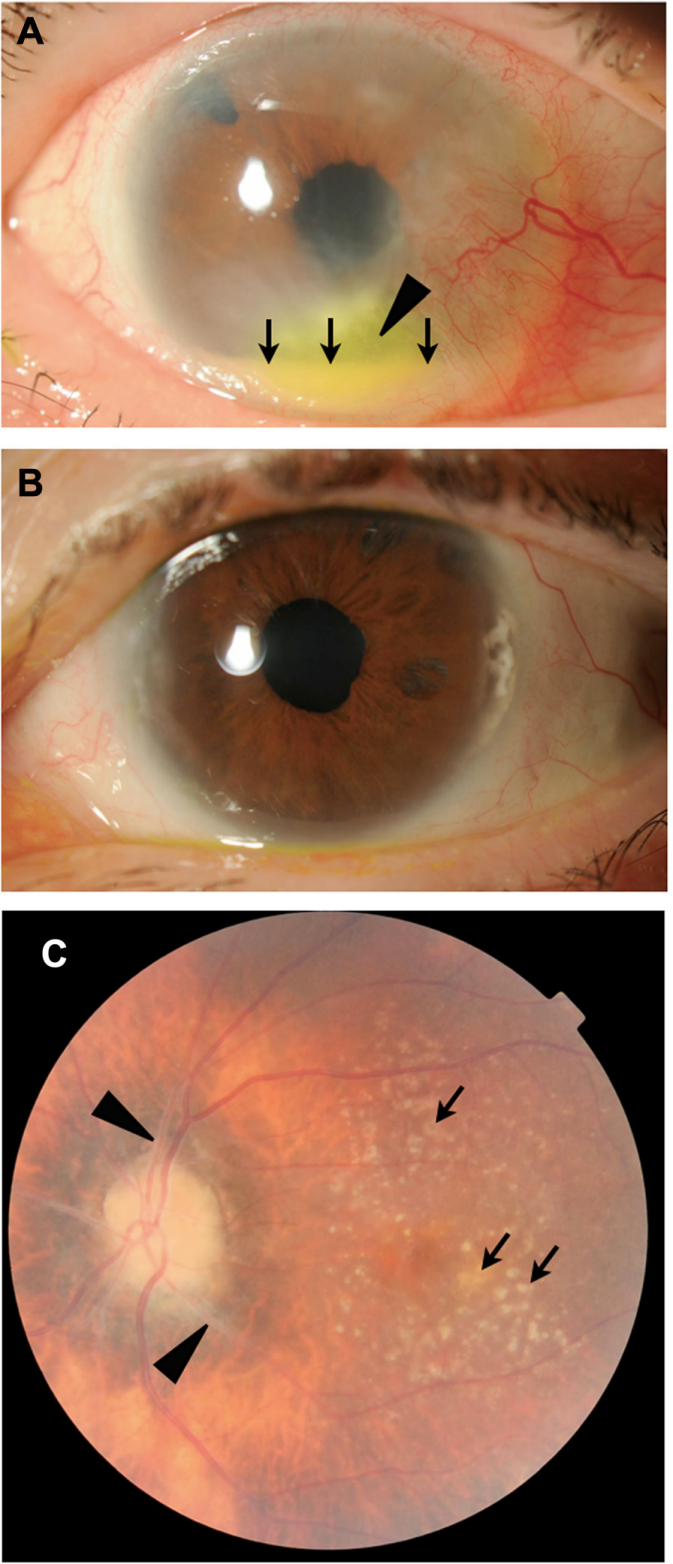
Initial examination of a 64-year-old female referred with ulcerative keratitis and anterior chamber inflammation. **a** Slit lamp biomicroscopy of the anterior segment of the right pseudophakic eye (visual acuity 20/400) showed diffuse conjunctival injection, pterygium-like corneal invasion of the conjunctival tissue, and the presence of corneal infiltrates with epithelial erosion (*arrowhead*). Marked cells and flares with hypopyon (*arrows*) were also present in the anterior chamber of the right eye. **b** Slit lamp biomicroscopy of the anterior segment of the left pseudophakic eye (visual acuity 20/22) appeared normal. **c** Fundus examination of the left eye showed a pale optic disc, sheathing of the retinal arteries (*arrowheads*) and yellowish deposits in the posterior pole (*arrows*)

At the patient’s initial visit, a clinical examination confirmed the presence of a rash on her body with a saddle nose and clubbed fingers. Biological inflammatory markers, including the white blood cell count and C-reactive protein (CRP) level, were high. The patient’s visual acuity was 20/400 in the right eye and 20/22 in the left eye, and the intraocular pressure was 11 mmHg in both eyes. Slit lamp biomicroscopy of the right pseudophakic eye showed diffuse conjunctival injection, pterygium-like corneal invasion of the conjunctival tissue, and the presence of corneal infiltrates with epithelial erosion (Fig. 1a). Marked cell and flare with hypopyon were also present in the anterior chamber of the right eye. An opening existed from previous laser iridectomy. The details of the right fundus were obscured by corneal opacity and anterior inflammation. Although the anterior segment of the left pseudophakic eye was normal, a fundus examination showed a pale optic disc, sheathing of the retinal arteries, and yellowish deposits in the posterior pole (Fig. 1b, c).

**Fig. 2:**
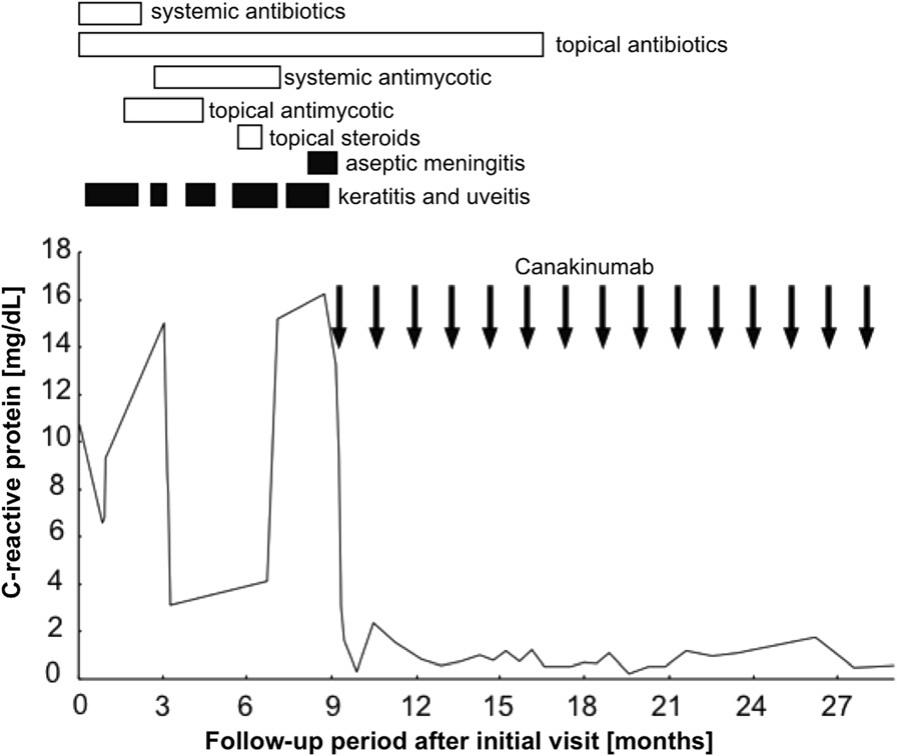
Schematic representation showing clinical course and treatment regime. Duration of medication (*open bars*) and patient’s symptoms (*closed bars*) showing that after initiating canakinumab therapy (*arrows*), all physical symptoms (rash, arthralgia, aseptic meningitis, and fever) and ophthalmic symptoms (conjunctival injection, ulcerative keratitis, anterior chamber inflammation, and hypopyon) were resolved within a few days. In addition, the level of C-reactive protein (CRP) normalized and stabilized. No recurrence or adverse events were observed during a follow-up period of 18 months

Based on the observations above, we considered the possibility of a corneal bacterial and/or fungal infection. The patient was therefore treated with topical and systemic antibiotics (eyedrops containing 0.5 % levofloxacin, 0.3 % gatifloxacin, 0.5 % cefmenoxime, and 0.3 % gentamicin and intravenous flomoxef at 2 g/day) and also with antifungal drugs (1 % pimaricin ointment and oral itraconazole at 200 mg/day). She received these medications as well as topical corticosteroid therapy (0.1 % betamethasone and 0.1 % fluorometholone eyedrops) for 9 months. However, this did not completely resolve the problem and repeated exacerbations of anterior inflammation with ulcerative keratitis continued. During the course of this treatment, we carried out microbiological investigations of corneal scrapes, virological investigations for varicella zoster, herpes simplex virus, and cytomegalovirus in the aqueous humor and tests for HLA-B51. However, all tests were negative. During this period, the patient experienced one episode of aseptic meningitis (Fig. 2). In addition, the urticaria-like rash on her body persisted and her CRP level remained elevated.

The presence of the urticaria-like rash, fever, arthralgia, and both her past history and her family history led us to consider a CAPS diagnosis. Therefore, a genetic analysis was carried out, and this detected a heterozygous, germline p.Asp303Asn mutation in the *NLRP3* gene in both the patient and her son, confirming a diagnosis of CAPS. Based on her severe physical and ophthalmic manifestations, including chronic meningitis and posterior segment involvement in the left eye, a diagnosis of CINCA/NOMID syndrome was made. The patient was therefore started on a course of 150 mg canakinumab (Ilaris; Novartis Pharma AG, Basel, Switzerland) given subcutaneously once every 6 weeks. All of the patient’s physical symptoms (rash, arthralgia, aseptic meningitis, and fever) and ophthalmic symptoms (conjunctival injection, ulcerative keratitis, anterior chamber inflammation, and hypopyon) resolved within a few days. In addition, the patient’s CRP level normalized and stabilized (Fig. 2).

**Fig. 3:**
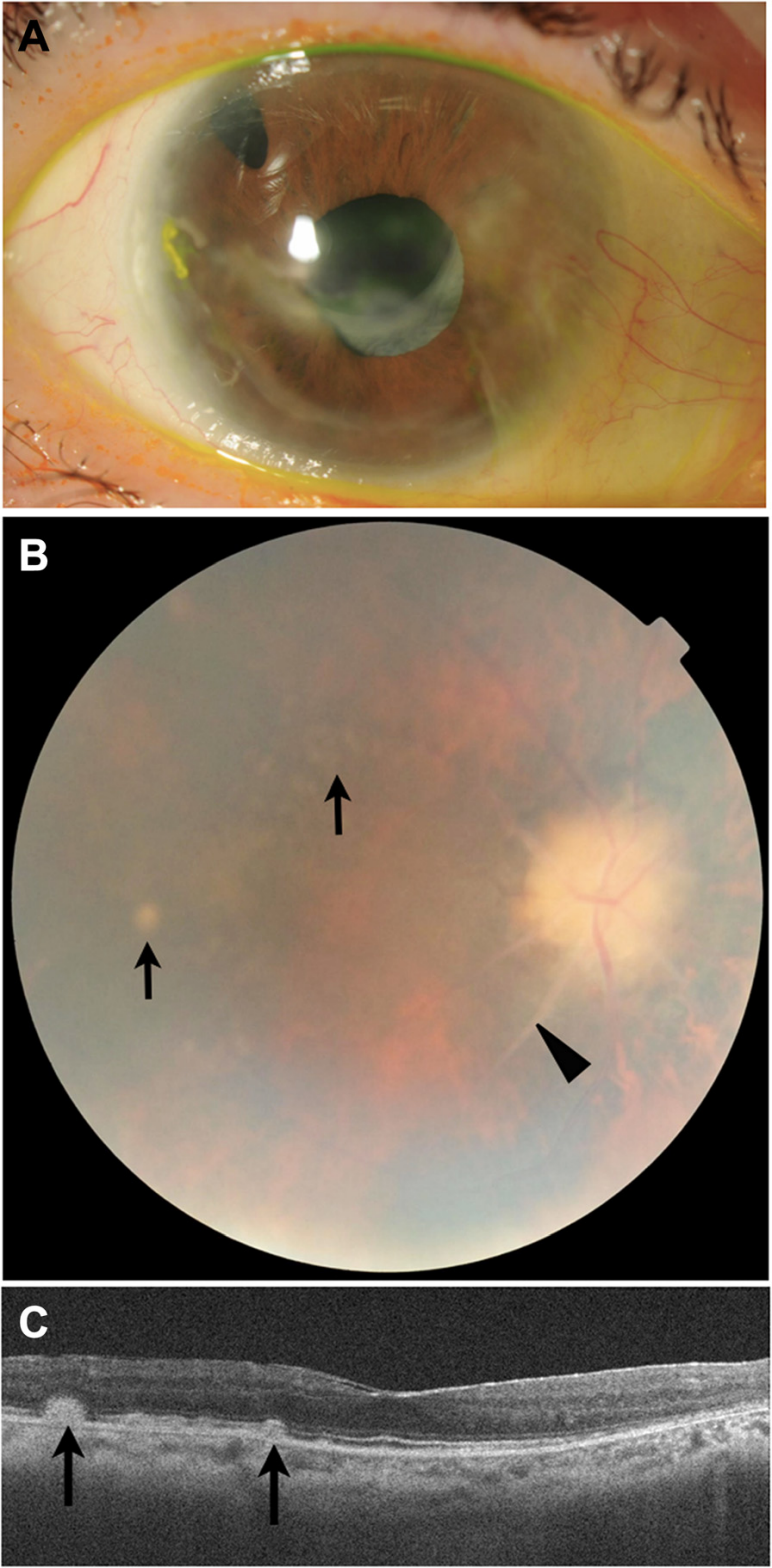
Ophthalmic findings in the right eye after canakinumab therapy. **a** Anterior inflammation (conjunctival injection, ulcerative keratitis, anterior chamber inflammation, and hypopyon) remained resolved 6 months after the initiation of canakinumab therapy. **b** Fundus examination of the right eye showed a pale optic disc with peripapillar sheath-like fibrosis involving the retinal vessels (*arrowhead*) and yellowish deposits in the posterior pole (*arrows*). **c** Spectral-domain optical coherence tomography showed intact ellipsoid zone and drusen-like subretinal deposits (*arrows*) internal to the retinal pigment epithelium

After the anterior inflammation had resolved, we conducted a fundus examination, which showed that the right eye was more severely affected than the left eye, with a pale optic disc, peripapillar sheath-like fibrosis involving the retinal vessels, and yellowish deposits in the mid-periphery (Fig. 3a, b). Spectral-domain optical coherence tomography (Cirrus; Carl Zeiss Meditec Inc., Dublin, CA) showed an intact ellipsoid zone and drusen-like subretinal deposits internal to the retinal pigment epithelium (Fig. 3c). At the patient’s last visit, which was 18 months after the first treatment with canakinumab, her visual acuity was 20/66 in the right eye and 20/20 in the left eye and slit lamp biomicroscopy showed no sign of anterior inflammation in the right eye. No adverse events had been documented during the follow-up period.

### Discussion

In this report, we have described a case of CINCA/NOMID syndrome-related stromal keratitis and uveitis in an adult female, which was successfully treated with canakinumab. To the best of our knowledge, this is the first report of CINCA/NOMID syndrome, which describes the ophthalmic findings before and after the canakinumab treatment in detail. In most of the previous reports describing the efficacy of canakinumab in patients with CAPS, conjunctivitis has been the only end point used to assess ocular manifestations [13, 15]. Therefore, the efficacy of canakinumab for a variety of ophthalmic symptoms had remained unclear. In the patient we have described in this report, we found that canakinumab therapy led to a marked improvement, not only in systemic inflammation but also in ophthalmic manifestations such as recurrent stromal keratitis and anterior uveitis. We were surprised that the subcutaneous injection of canakinumab resolved the ophthalmic inflammation so dramatically, in spite of the presence of blood-retina and blood-aqueous barriers in the eye. Canakinumab has been reported to induce adverse events, such as nasopharyngitis, upper respiratory infections, and gastroenteritis, presumably due to the effects of blocking the actions of the proinflammatory cytokine IL-1β [13, 15]. However, no side effects were observed during the follow-up period in this case. Further observation will be needed to check the long-term efficacy of canakinumab.

This case indicates that it is important to make a diagnosis and start treatment with canakinumab as early as possible to prevent irreversible ocular complications. In our patient, the visual acuity of the right eye did not completely recover, as the delay in diagnosis resulted in post-inflammatory optic disc atrophy and stromal opacification. It has been only 13 years since the mutation of CIAS1 was discovered in CAPS patient. Although the discovery has made much progress in pathophysiologic and therapeutic understanding of CAPS, clear diagnostic criteria have not been yet established. In addition, CAPS is a very rare disease; for example, CINCA/NOMID syndrome has a prevalence of 1/4,000,000 in Japan [16]. These conditions can cause our lack of awareness of the disease, resulting in delayed diagnosis and treatment. We think it is important to consider CAPS including CINCA/NOMID syndrome in the differential diagnosis for patients who present with periodic fever, urticaria-like rashes, meningitis, articular manifestations, and ocular inflammation.

In conclusion, our case report indicates canakinumab is effective in CINCANOMID syndrome, not only for systemic inflammation but also for ophthalmic involvement, such as recurrent stromal keratitis and anterior uveitis. Further prospective studies will be required to determine the efficacy of canakinumab, including as many patients as possible with this rare disease.

### Consent

Written informed consent was obtained from the patient for publication of this case report and accompanying images. A copy of the written consent is available for review by the Editor-in-Chief of this journal.

## Abbreviations

CAPS: cryopyrin-associated periodic syndrome
CINCA: chronic infantile neurologic, cutaneous and articular
CRP: C-reactive protein
IL-1β: interleukin-1β
NOMID: neonatal-onset multisystem inflammatory disease
